# GECO: A Real-Time Computer Vision-Assisted Gesture Controller for Advanced IoT Home System

**DOI:** 10.3390/s26010061

**Published:** 2025-12-21

**Authors:** Murilo C. Lopes, Paula A. Silva, Ludwing Marenco, Evandro C. Vilas Boas, João G. A. de Carvalho, Cristiane A. Ferreira, André L. O. Carvalho, Cristiani V. R. Guimarães, Guilherme P. Aquino, Felipe A. P. de Figueiredo

**Affiliations:** 1 Inatel Competence Center (ICC), National Institute of Telecommunications, João de Camargo Avenue 510, P.O. Box 05, Santa Rita do Sapucaí 37540-000, MG, Brazil; murilo.lopes@inatel.br (M.C.L.); cristiane.andare@inatel.br (C.A.F.); andreluiz.carvalho@inatel.br (A.L.O.C.); cristiani.vilela@inatel.br (C.V.R.G.); 2 Critical Telecommunications and IoT Infrastructure Laboratory (CTIoT Lab.) and Cyber Security Center (Centro de Segurança Cibernética do Inatel—CxSC Telecom), Department of Telecommunication, National Institute of Telecommunications, João de Camargo Avenue 510, P.O. Box 05, Santa Rita do Sapucaí 37540-000, MG, Brazil; paulaapsilva@inatel.br (P.A.S.); joao.jg@ges.inatel.br (J.G.A.d.C.); guilhermeaquino@inatel.br (G.P.A.); 3 Devlane, Luis Alberto de Herrera 1248, Montevideo 11300, Uruguay; ludwing.marenco@devlane.com; 4 Future Technologies Development Center, Rua Doutor Gilberto Studart 55, Fortaleza 60192-105, CE, Brazil; 5 Wireless and Artificial Intelligence Laboratory (WAI Lab.), Departament of Telecommunication, National Institute of Telecommunications, João de Camargo Avenue 510, P.O. Box 05, Santa Rita do Sapucaí 37540-000, MG, Brazil; felipe.figueiredo@inatel.br

**Keywords:** gesture control, internet of things, home automation, MediaPipe framework, smart home

## Abstract

This paper introduces GECO, a real-time, computer vision-assisted gesture controller for IoT-based smart home systems. The platform uses a markerless MediaPipe interface that combines gesture-driven navigation and command execution, enabling intuitive control of multiple domestic devices. The system integrates binary and analog gestures, such as continuous light dimming based on thumb–index angles, while operating on-device through a private MQTT network. Technical evaluations across multiple Android devices have demonstrated ultra-low latency times (<50 ms), enabling real-time responsiveness. A user experience study with seventeen participants reported high intuitiveness (9.5/10), gesture accuracy (9.2/10), and perceived inclusivity, mainly for individuals with speech impairments and low technological literacy. These results position GECO as a lightweight, accessible, and privacy-preserving interaction framework, advancing the integration of artificial intelligence and IoT within smart home environments.

## 1. Introduction

Internet of Things (IoT) solutions enable real-time data monitoring and remote process control through data-driven decision-making [1,2,3]. Advances in wireless communication standards, such as fifth-generation mobile networks (5G), Wi-Fi 6, and Low-Power Wide-Area Networks (LPWANs), have provided higher bandwidth, lower latency, and improved energy efficiency, fostering the adoption of IoT technologies and meeting the increasing data demands of interconnected devices [4,5,6,7]. As a result, IoT has driven the development of smart city services and applications, accelerating digital transformation across multiple domains [1,2,3,8,9]. The urban ecosystem is a complex network designed to meet fundamental human needs, such as food, housing, sanitation, health, public safety, and leisure. It integrates areas essential to socio-economic development, including civil infrastructure, transportation, economy, industry, agriculture, energy, health, and governance [8,9,10,11,12,13]. Effective urban planning relies on understanding and managing these interrelated systems.

Reliable infrastructure, affordable housing, and efficient transportation form the foundation of smart city environments. Economic growth, sustainable industries, and a diversified energy matrix strengthen urban resilience and development. Accessible healthcare improves quality of life, while transparent and participatory governance fosters policies aligned with public interests. Within this framework, citizens act as active contributors to smart city development rather than mere beneficiaries. Technological advancements optimize city operations and accelerate urban development through digital transformation. These innovations influence all sectors, including infrastructure, transportation, energy, health, and education, shaping social and cultural dynamics [10,11,12,13]. The integration of IoT with Big Data, cloud computing, 5G, and artificial intelligence (AI) enables real-time data analysis, promoting efficient and adaptive urban management, enhanced quality of life, and economic growth [4,7,14,15,16,17]. Moreover, these technologies support sustainability by encouraging renewable energy adoption, reducing waste, and minimizing environmental impact.

Civil infrastructure and housing encompass key urban elements, including residential buildings, houses, and the management of public services related to culture, education, and tourism. The adoption of IoT-enabled solutions has driven the development of smart buildings, smart homes, and smart living services [8,9,10,11,12]. Smart buildings integrate automation and management systems for security, access control, video surveillance, and human activity monitoring. They also automate energy, gas, and water distribution systems, as well as fire detection, gas leak monitoring, and structural health assessment. These solutions depend on technologies that collect, store, and process data from daily building operations.

Smart homes represent a subset of smart infrastructures, focusing on integrating technological devices within domestic environments. While smart buildings provide scalable and standardized solutions for multiple residences, smart homes target individual households [1,2,3]. In a smart home environment, connected devices and automation systems enable the monitoring and control of temperature, lighting, and security, enhancing comfort, reducing energy consumption, and improving resource efficiency. Products such as Echo Dot, Nest Mini, and HomePod function as voice-controlled assistants, supporting the integration of IoT devices like smart lights, plugs, air conditioners, locks, and curtains, which can be managed via mobile applications or voice commands [18].

However, as smart home ecosystems continue to evolve, the next stage of innovation depends on integrating AI-based solutions [19,20,21]. Current IoT devices enable remote monitoring and control through predefined commands, while future smart homes are expected to incorporate adaptive and predictive capabilities to anticipate user needs and autonomously manage domestic environments. AI algorithms analyze behavior patterns, environmental conditions, and device usage to optimize energy consumption, enhance security, personalize user experiences, and enable predictive maintenance. Furthermore, AI-enabled smart home systems promote inclusivity by adapting interfaces and functionalities to meet the needs of diverse users, including the elderly and people with disabilities [22,23,24]. Through voice recognition, gesture control, and contextual awareness, AI-powered smart homes enhance accessibility, independence, and comfort, transforming residences into dynamic, self-learning environments that continuously improve over time.

Based on this context, this work presents GECO, an intuitive gesture-based controller that leverages real-time computer vision to operate IoT devices within smart home environments. GECO enables non-contact interaction through hand-tracking, supporting both discrete commands and continuous analog actions such as intensity adjustment. The platform prioritizes responsiveness and accessibility while maintaining fully local processing, avoiding dependencies on external cloud services. In addition, GECO promotes technological inclusion by enabling individuals with speech impairments, such as nonverbal users, to interact with smart environments, while also supporting elderly and non-technical users. The system can further be extended to domains beyond home automation, enhancing human–machine interaction in sectors such as industrial automation. The main contributions of this work are summarized as follows:

- Development of GECO, a markerless gesture controller for IoT smart homes integrating MediaPipe-based hand tracking with local device control.
- Design of a two-level interaction model in which the right hand enables on-screen navigation and the left hand controls device states and analog adjustments.
- Implementation of continuous gesture-based modulation, such as light dimming using thumb–index angles, enabling more natural device interaction.
- Performance validation across multiple devices demonstrating real-time responsiveness and consistent user experience, supported by quantitative latency analysis (<50 ms) and empirical cumulative distribution function (ECDF) modeling.

The remainder of this work is organized as follows. Section 2 reviews related studies on gesture control and technological developments, comparing them with the GECO platform to highlight its advancements. Section 3 presents the computer vision-assisted gesture control platform and identifies the enabling technologies within each IoT layer. Section 4 describes the GECO implementation, and Section 5 discusses the testing procedures, including command latency across different devices and user experiences. Finally, Section 6 provides the main conclusions and outlines future research directions.

## 2. Related Works

Automation modules were the first residential device solutions, designed to minimize human intervention in domestic environments. Over time, smart devices emerged to offer more accessible and customizable interactions for household users. Smart home technologies have since evolved considerably, driven by the growing IoT ecosystem and the adoption of enabling technologies such as 5G networks and AI. Currently, users can equip their homes with integrated commercial solutions that form comprehensive IoT control systems. User interfaces have progressed from touchscreen displays and mobile applications to voice-controlled assistants capable of direct interaction with users. However, despite the availability of commercial IoT solutions, users still encounter challenges in device installation and integration, motivating the development of unified framework projects.

An IoT-centric framework based on a user interface has been proposed to integrate applications, servers, databases, devices, and APIs, enabling a scalable IoT architecture [25]. The implementation demonstrated the framework’s scalability, modularity, and interoperability. Within this context, a smart campus solution integrated IoT devices to collect and record real-time data, supporting continuous analysis, emergency response planning, and data-driven decision-making [26]. Similarly, an IoT-enabled laboratory concept was implemented to create a smart learning and teaching environment [27]. This smart laboratory replicates real intelligent environments monitored by sensors that record data to support statistical modeling. Experiments conducted in this setting focused on data collection related to air quality, waste management, lighting, and noise pollution. Another smart laboratory initiative combined IoT devices with voice, text, and visual dashboards to automate environmental control and assist daily research activities [28]. Additionally, a Smart Lab platform was developed featuring a mobile application with a user interface that supports touchscreen commands and voice-assisted interactions [2]. This user-centric application enables command execution through predefined touchscreen buttons or via a virtual assistant powered by Natural Language Processing (NLP) and Natural Language Understanding (NLU) machine learning (ML) algorithms.

The aforementioned IoT-centric frameworks enable the evolution of commercial solutions from simple smart devices to comprehensive IoT home control systems. These frameworks rely on user interfaces implemented through mobile and web applications, some of which integrate virtual assistants powered by NLP and NLU. Despite the progress in incorporating ML capabilities and achieving interoperability across IoT layers, these projects still face challenges related to user-friendliness, scalability, and adaptability [29,30]. Hence, gesture-controlled interfaces have emerged as a seamless alternative for device interaction, complementing the modes explored by existing solutions. These controllers employ computer vision to track user commands through event-based cameras [29,30,31]. Their applications have recently expanded across various IoT domains, including industrial interfaces [31,32], e-health solutions [33,34,35], and smart home control systems [36,37,38,39,40,41,42,43,44].

In addition to general-purpose smart home automation, recent research has advanced target-oriented sensing, enabling precise monitoring and fine-grained control of critical parameters in specialized environments. Systems such as WiFi-based healthcare sensing architectures demonstrate how ambient wireless signals can be repurposed to infer subtle human activities, micro-gestures, or physiological variations without requiring wearable devices [45]. Target-oriented WiFi sensing, in particular, leverages beamforming and interference mitigation to enable robust respiration monitoring for elderly or medically vulnerable users [46]. These works highlight the potential of combining high-resolution sensing with assistive applications. From this perspective, GECO’s real-time analog gesture capabilities, such as continuous thumb–index angle estimation, could support adaptive environmental adjustments or personalized assistance functions in healthcare-oriented settings. Such extensions enhance inclusivity by supporting populations with motor limitations, elderly individuals, or speech-impaired users, while complementing targeted sensing systems in next-generation ambient intelligence applications.

Regarding IoT home system control, hand gesture recognition enables intuitive and non-contact interaction with smart devices. Related works have explored various sensing modalities, classification algorithms, and application scenarios to enhance gesture-based interfaces. For instance, an advanced gesture recognition method combining the fractional Fourier transform (FrFT) with a relevance vector machine (RVM) was proposed for smart home appliance control [36]. This approach employs millimeter-wave radar to achieve recognition rates above 98%, effectively addressing challenges in feature extraction and real-time responsiveness. By integrating optimized feature selection and classification, it outperforms traditional ML and deep learning techniques. Ameliasari et al. [39] investigated the use of inertial measurement units (IMUs) embedded in smartwatches to detect hand gestures for smart lighting control. The support vector machine (SVM) evaluation, combined with feature selection techniques, showed that reducing the feature set can improve accuracy. Additionally, a system leveraging Google MediaPipe for real-time hand tracking and gesture recognition was developed to enhance the accessibility of smart home controls, particularly for elderly and disabled users [42]. By computing angles between hand key points to control IoT-enabled devices, the system emphasizes cost efficiency and user convenience while maintaining high precision and recall.

A scalable system named SeleCon, which integrates ultra-wideband (UWB) positioning with hand gesture recognition for device selection and control in IoT environments, was introduced in [37]. SeleCon enables reliable gesture recognition and precise device targeting while minimizing energy consumption by combining inertial sensing with UWB ranging, addressing scalability and usability challenges in densely populated smart homes. Alabdullah et al. proposed a markerless hand gesture recognition method that fuses multiple features and employs a recurrent neural network (RNN) classifier for smart home automation [40]. The system processes dynamic gestures extracted from video frames, achieving high accuracy across several public datasets and providing a robust solution that does not depend on specialized hardware. Similarly, Panagiotou et al. explored multidisciplinary ML techniques for gesture recognition to assist people with disabilities [38]. The authors conducted a comparative analysis of IMU-based classification, edge device learning, and computer vision approaches, highlighting the potential of integrated models such as MoveNet combined with convolutional neural networks (CNNs) for accurate, real-time gesture recognition in ambient assisted living (AAL) environments.

Fatima et al. developed an American Sign Language (ASL) recognition system based on computer vision and CNNs to control smart home appliances [41]. The study focused on promoting inclusivity for individuals with auditory impairments, demonstrating that gesture recognition can enhance communication and autonomy in smart home contexts. Additionally, an architecture integrating gesture recognition with oneM2M standards for smart home environments was proposed, analyzing both user-dependent and user-independent models [44]. The study evaluated logistic regression and decision tree classifiers for gesture control tasks, emphasizing the trade-offs between model complexity, server resources, and latency when deploying gesture-based control systems at scale. Wearable sensor-based solutions, such as smartwatches equipped with IMUs, have also achieved good accuracy for gesture recognition [37,39], but these approaches require physical devices and offer limited intuitiveness. In contrast, vision-based systems like those in [40,42] enable hands-free interaction but depend on cloud-based processing or only support binary controls.

The proposed GECO platform addresses these limitations by combining markerless, real-time gesture recognition with support for continuous actions, while relying on fully local processing to reduce latency and enhance privacy. Unlike previous solutions that focus on gesture classification or device actuation in isolation [36,41], GECO integrates these components into a unified interaction model tailored for everyday smart home use. Table 1 summarizes this comparison by highlighting differences across gesture sensing, interaction models, and control architectures.

## 3. Computer Vision-Assisted Gesture Controller IoT Platform

An IoT solution integrates sensors, actuators, and microcontrollers that communicate through standardized protocols with databases and user applications. The system architecture is typically organized into a five-layer vertical structure [1,2]. At the perception/actuation layer, sensors capture environmental or biological stimuli, while actuators modify the surroundings according to microcontroller commands. Data is transmitted to higher layers using wireless communication protocols selected based on IoT constraints such as bandwidth, coverage, and energy consumption [1,2,47,48,49]. The middleware layer processes and stores data using servers and databases, such as MySQL or NoSQL, enabling device communication through client-server or publish/subscribe models. The application layer provides user interfaces for monitoring and control, whereas the business layer employs statistical analysis to support investment decisions and future service development [1,2].

**Figure 1:**
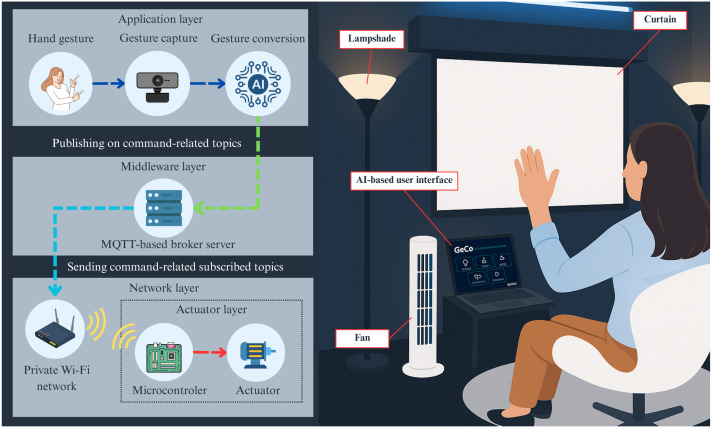
Architecture of GECO: Computer vision-assisted gesture controller platform for IoT home system control.

The proposed computer vision-assisted gesture-controlled IoT platform is based on the five-layer vertical architecture, although not all layers are fully implemented. As illustrated in Figure 1, GECO operates as a consumer IoT platform similar to other commercial solutions, providing a private network for end users and functioning within a confined environment. Following a bottom-up perspective, the platform integrates microcontrollers and actuators within hardware control modules, a private Wi-Fi network, a publish/subscribe broker server based on the MQTT protocol, and a gesture-controlled user interface powered by computer vision. The actuators were selected according to the proposed scenario to demonstrate the system’s capabilities, including a motor for curtain control and an electrical relay for switching fans and lamps on and off. The platform focuses on actuators, adopting a user-centric approach that enables individuals to interact with and adjust their environment according to personal preferences. This remote-control IoT configuration aligns with the use case defined in ITU-T Recommendation Y.2066 (https://www.itu.int/rec/T-REC-Y.2066/en) (accessed on 20 July 2025), which encompasses home automation [2]. Furthermore, the microcontroller is configured to communicate with upper layers through a private Wi-Fi network, allowing it to control actuators based on received command information.

The private network was designed to enable communication among the components of the IoT platform. Communication is established using the Wi-Fi protocol and managed through MQTT. The MQTT publisher client corresponds to the device running the gesture controller application, which can be a smartphone, tablet, or notebook. The MQTT subscriber clients consist of two ESP8266 Wi-Fi microcontrollers: one controlling the motorized curtain and the other managing the fans and lamps. The MQTT broker operates on a virtual machine configured with a dedicated IP address to ensure that the IoT network remains private and accessible only to authorized devices. The Mosquitto MQTT client library was used for publishing and subscribing to messages. Each device in the platform is assigned a unique MQTT topic, organized hierarchically to differentiate between functionalities. A total of seven topics were implemented: one for the curtain, two for the fans, two for the lamps, and two additional topics for lamps with adjustable intensity.

The motorized curtain was equipped with a 12 V DC motor and an eight-tooth gear, both connected to an H-bridge driver. The driver interfaces with two digital pins of the Wi-Fi microcontroller, allowing control of the motor’s rotation direction and enabling the curtain to move upward or downward. Published messages for the curtain topic are “true” and “false,” where “true” initiates movement and “false” stops the motor. Based on the previously stored state in an external variable, the Wi-Fi microcontroller determines whether the curtain should move up or down. Two ultrasonic sensors were installed at predefined minimum and maximum heights to prevent the motor from exceeding the curtain’s movement limits. These sensors ensure that the curtain stops automatically at the upper and lower boundaries.

The second microcontroller controls commercial fans and lamps through an electrical relay, as these devices operate with high-voltage current. The relay provides safe switching between on and off states. Additionally, two lamps with adjustable intensity were implemented using 7-bit addressable RGB LEDs. For these lamps, the published messages correspond to integer values ranging from 0 to 100, where 0 represents no intensity and 100 represents maximum brightness. This value is mapped to a range between 0 and 255 and used as the brightness parameter in the CHSV (Hue-Saturation-Value) function of the FastLED library. The hue and saturation values are fixed at 75 and 0, respectively, ensuring consistent color output. Therefore, the final function is expressed as CHSV (75, 0, brg), where brg represents the mapped brightness value.

The gesture controller interface was implemented in two phases. The first phase consisted of deploying the software solution, and the second focused on the computer vision component, specifically hand landmark detection and gesture classification. The software deployment includes a mobile application developed in Kotlin version 1.7.10, which serves as the user interface. Gesture classification was implemented using the MediaPipe framework [50]. Details about the GECO user interface are presented in Section 4.

## 4. Computer Vision-Assisted Gesture Controller Implementation

This section presents the GECO implementation, focusing on the user interface and the ML-based pipeline developed using the MediaPipe framework [50]. The ML pipeline comprises a set of models for hand tracking, including left–right hand classification, hand-landmark detection and gesture classfiication. While several other hand-tracking models and frameworks exist [51,52,53], MediaPipe was chosen due to its lightweight and highly optimized design, which enables fast, real-time hand tracking directly on mobile devices. Unlike alternative solutions, it does not require specialized hardware or complex model-deployment procedures, making it well-suited for mobile applications.

**Figure 2:**
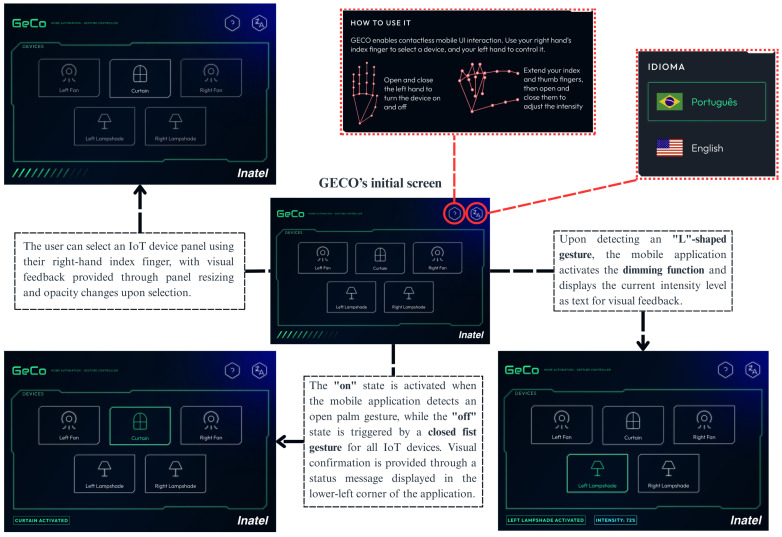
Implemented user interface for the computer vision-based gesture controller. The screen navigation is based on a two-level interaction, in which the right hand navigates among devices and interface instructions, and the left-hand gesture controls the device state (“on” and “off”), including dimming via finger-angle estimation.

Figure 2 illustrates the user interface, which features a visual layout composed of five rectangular sub-panels. Each panel represents an IoT device, as defined in Section 3, that can be controlled through gestures, eliminating the need for touchscreen interaction. Additionally, two hexagon-shaped panels are located in the upper right corner of the interface. These panels open alert dialog windows, with the left panel displaying usage instructions for the gesture controller and the right panel allowing users to switch the interface language between English and Portuguese.

Real-time gesture detection is enabled using MediaPipe’s precompiled Android SDK for hand tracking, integrated into the Kotlin application through a dedicated processing loop that continuously receives frames from the device’s camera. When the application is launched, the function containing the Mediapipe hand tracking pipeline loads three models into memory: the palm detection model, the hand landmark model, and the gesture classification model, all required for gesture recognition. The load model step is carried out by setting the default minimum confidence thresholds for hand detection, presence, and tracking and this process occurs only once per session. Models architectures, its training data and prediction return values for each model is succinctly exposed as follows.

The palm detector model is based on a single-shot detection architecture optimized for real-time performance on mobile devices [54]. Rather than detecting fully articulated hands, the model focuses on simplified square palm regions, which reduces complexity and improves robustness in scenarios involving occlusion or overlapping hands. Training data consists of approximately 6000 real-world images encompassing diverse geographies, skin tones, lighting conditions, backgrounds, and hand appearances. To mitigate the class imbalance inherent in dense anchor-based detection, focal loss was employed during training. Reported average precision of model is 95.7% [54]. Once a palm is detected, the model outputs an oriented bounding box, enabling the extraction of a well-aligned cropped region for subsequent landmark tracking.

The hand-landmark model is a regression network trained to predict 21 2D keypoints along with a relative depth value for each joint, resulting in a 2.5D representation of the hand [50]. In addition, the model outputs a handedness classification (left or right) and a confidence score indicating whether a properly aligned hand is present in the cropped region. The training dataset includes both real and synthetic images. The real-world portion consists of approximately 16,000 manually annotated images containing 2D landmarks and handedness labels. The synthetic dataset comprises around 100,000 rendered images, introducing extensive variation by animating a realistic 3D hand model into diverse poses, applying different lighting conditions, and rendering from multiple camera viewpoints against randomized backgrounds. hand-landmark model achieved a mean normalized absolute error (MNAE) of about 10.1% (±1.7% standard deviation) across a geographically diverse validation set, indicating sub-centimeter landmark accuracy normalized by palm size which is generally sufficient for reliable recognition of coarse hand gestures and directional controls, making it well-suited for real-time control tasks [55].

The gesture classification model operates on the output of the hand-landmark model rather than raw RGB input, it takes the 21 hand landmarks as input and classifies the hand pose into one of a fixed set of gesture categories, without processing any pixel data. By default, the recognizer classifies gestures into one of eight predefined categories: Unrecognized Gesture, Closed Fist, Open Palm, Pointing Up, Thumbs Down, Thumbs Up, Victory, and Love. This model is organized as a two-stage neural-network pipeline. first stage is an embedding network converts the landmark coordinates into a compact feature vector, then in the second stage a lightweight classification network maps that embedding to one of the predefined gestures. The gesture classification model reported a high weighted-average SS F1-score of 95.5% across varied perceived skin-tone groups and 93.9% across perceived gender-expression groups, with no observed systematic bias across these demographics [56]. This model outputs a vector of probabilities over these gesture classes. It is important to note that gesture recognition is not limited to the eight predefined gestures mentioned earlier. The system can be expanded to support additional gestures either by training a custom model using MediaPipe’s available training tools or by programmatically defining new gestures based on specific combinations of landmark coordinates.

The gesture controller procedure works as provided in Algorithm 1. Once the models are loaded, each incoming frame from the input stream is processed by the palm detection model to localize the hand region. Within this region of interest, the hand landmark model estimates 21 two-dimensional landmark coordinates along with handedness information. These landmarks are subsequently passed to the gesture classification model, which assigns them to a predefined gesture label. To enable a natural and touch-free interaction with the mobile app, the recognized handedness is used to differentiate two complementary interaction levels: right-hand gestures control user navigation within the graphical interface, while left-hand gestures are exclusively reserved for issuing IoT device state-change commands. This design aims to ensure that UI navigation remains independent from device control actions, minimizing accidental activations and improving usability.
**Algorithm 1**: Computer Vision-Assisted Gesture Controller.
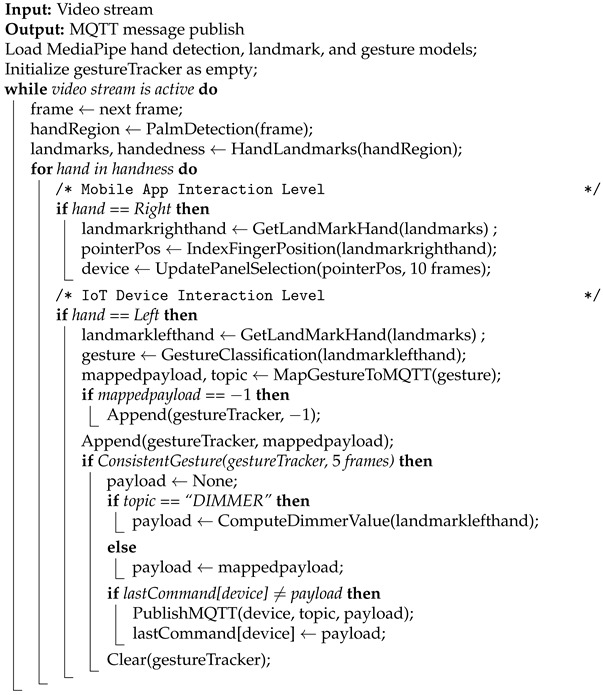


User navigation is driven by the two-dimensional coordinate of the right-hand index finger tip, represented as landmark ID 8 in the MediaPipe hand-landmark topology [57], which acts as a virtual pointer to explore and select panels within the graphical interface. The mobile application registers a panel selection only when the index finger remains within the panel boundaries for more than 10 consecutive frames, corresponding to an average time of 33ms in a 30 fps camera. The minimum number of frames for panel selection was set up experimentally. When one of the five central panels is selected, its size increases, while the other panels are scaled down, reducing their opacity, as shown in Figure 2. This approach follows UI/UX design principles to guide user attention and provide visual confirmation that an IoT device has been selected. The selected device is then stored in a variable for subsequent MQTT message construction. In contrast, selecting one of the two hexagon-shaped panels in the upper-right corner triggers a dialog box displaying either usage instructions or interface language options. These informational panels are designed solely for navigation purposes and are not used to generate MQTT messages.

The second level of interaction is responsible for controlling IoT device states based on left-hand gestures. Each of the five devices supports one main topic “turnon”, and state changes are encoded through a predefined gesture-to-topic mapping procedure. Only gestures explicitly linked to device control actions are converted into MQTT messages for transmission to the lower control layers. Any gesture outside this mapping receives a default code of −1, ensuring that unintentional or irrelevant hand movements do not trigger commands. Specifically, Open Palm and Closed Fist gestures are mapped to the payloads “true” and “false”, respectively. When one of these gestures is consistently recognized, the system publishes a message to the topic associated with the currently selected device and displays a notification in the lower-left region of the screen confirming the executed state change. For instance, a valid command to activate the left fan is expressed as “smarthome/leftfan/turnon: true”. In addition, the lamp supports an extended functionality that enables continuous adjustment of light intensity whenever its state is active. This adjustment is triggered by a custom “L”-shaped gesture, programmatically defined using geometric constraints between thumb and index finger landmarks. Specifically, the gesture is recognized when the following conditions are satisfied: the *y*-coordinate of the middle finger tip (landmark ID 12) is greater than that of its metacarpal (landmark ID 9); the *y*-coordinate of the ring finger tip (landmark ID 16) is greater than that of its metacarpal (landmark ID 13); and the *y*-coordinate of the pinky finger tip (landmark ID 20) is greater than that of its metacarpal (landmark ID 17). Once the gesture is detected, the light intensity value is computed from the angle formed by the thumb and index finger given by

(1) $$\theta =\arctan^{2}\left(\mathrm{length},\mathrm{base}\right)\times \frac{180}{\pi },$$

where lenght and base represent the distances between the tips of the thumb and index fingers and the distance between the tip of the thumb finger and the wrist, respectively. Angle θ is normalized using min-max normalization, with $\theta_{\min}=15$ and $\theta_{\max}=50$. $\theta_{\min}$ and $\theta_{\max}$ were determined by experimental testing.

The normalized angle is then multiplied by 100 to map it onto an intensity scale, which serves as the payload for the message. For example, a valid dimming command for the lamp takes the form “smarthome/lamp/dimmer: 87”, where 87 represents 87% of intensity. This dimming approach remains effective regardless of the distance between the left hand and the camera, as the mobile application only requires detection of the key-finger landmarks to compute intensity. The dimmer function is activated exclusively when the application detects an “L”-shaped gesture formed by the thumb and index finger of the left hand, preventing unnecessary intensity computations during user interaction. The interface provides visual feedback for this gesture by displaying an additional text element that indicates the current intensity level, as shown in Figure 2.

Finally, to ensure robust recognition of left-hand gestures and prevent erroneous topic mappings due to transient or noisy detections, a gesture-tracking mechanism was implemented. This tracker consists of a vector that stores the recognized gesture for each incoming frame. A gesture is only considered valid and allowed to trigger a topic mapping if it remains consistent for at least five consecutive frames, which corresponds to approximately 16.6ms at a 30 fps frame rate. After a gesture has successfully been published to its associated MQTT topic, the tracking vector is cleared, ensuring that the gesture must be reaffirmed before a new message can be generated. Additionally, to avoid unnecessary network communication, the system verifies whether the payload for the currently selected device has changed. If no change is detected, message publication is suppressed.

## 5. Technical and User Experience Discussion

**Figure 3:**
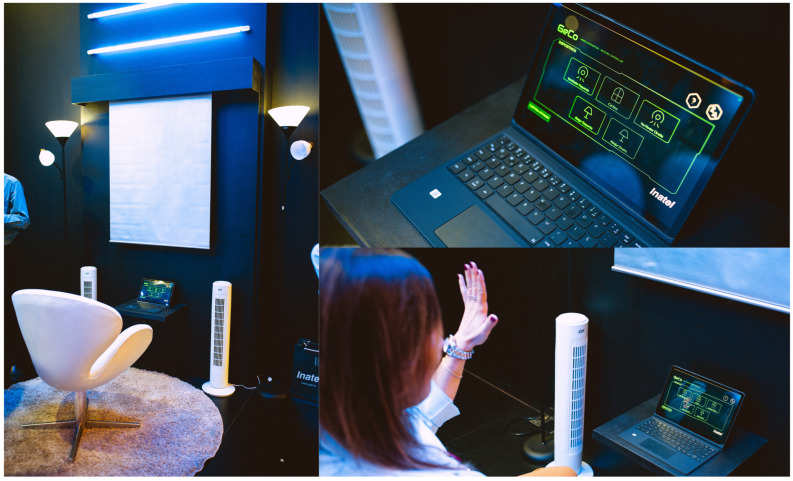
Demo version of GECO platform for IoT home system control.

The gesture controller platform was implemented in a demo version integrated with the IoT devices, as shown in Figure 3. The user interacts with GECO’s interface by positioning itself in front of a device running the gesture controller software. The right hand is used to select the target IoT device, guided by a red pointer displayed on the screen, while the left hand activates the command to change the device’s state. Selecting a device with the right hand configures the corresponding MQTT topic, whereas the left-hand gesture defines the message payload. This message is transmitted from the gesture controller software to the middleware layer over the private Wi-Fi network, processed by a microcontroller at the actuator layer, and triggers a change in the actuator’s state.

### 5.1. Technical Evaluation

A user experience experiment was conducted to evaluate the performance of the computer vision-assisted gesture controller. A single user, with prior knowledge of the platform, stood in front of GECO’s interface, selected a random device using their right hand, and performed successive command state changes with their left hand at 3-s intervals over a one-minute period. A timer placed in front of the mobile device helped user accurately time each gesture to trigger the state change of the selected IoT device. This experiment was repeated five times per device across four different commercial mobile devices. During each test, the inference time for gesture recognition was recorded, defined as the time required by the model to predict the user’s gesture. The average inference time was then calculated for each mobile device. The commercial mobile devices used in the user experience experiment are listed in Table 2. Additional devices running Android versions earlier than 9 were also tested, but the Kotlin application could not be successfully deployed due to compatibility issues.

**Figure 4:**
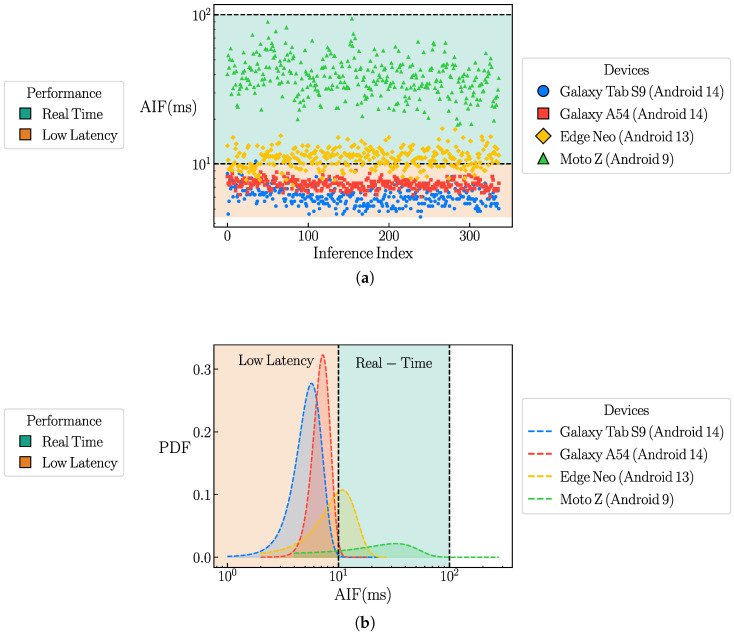
Performance analysis of the computer vision–assisted gesture controller across different mobile devices. (**a**) average inference time (AIF) values for each mobile device used and (**b**) empirical cumulative distribution function (ECDF) of the average inference time for each device.

Figure 4a presents the first 300 average inference time (AIF) values, measured in milliseconds, for each mobile device used in the experiment. The color and marker scheme for each device is detailed in the legend located at the center-right of the plot. Additionally, two shaded regions indicate performance zones: the lime-colored area represents the real-time response zone, corresponding to inference times between 10ms and 100ms, while the orange area highlights the ultra-low-latency zone, where inference times are below 10ms. A separate legend, positioned at the center-left of the plot, explains the color coding of these inference response zones.

The response time of the computer vision-assisted gesture controller is defined as the mean of the average inference times measured for each mobile device. These values were computed based on the empirical cumulative distribution function (ECDF) of the average inference time for each device. The resulting ECDFs were then fitted to normal distributions $N(\mu ,\sigma^{2})$ using the curve_fit function from the SciPy Python package [58]. Figure 4b presents the probability density functions (PDFs) of the fitted curves for all tested mobile devices. In this figure, the colored zones indicate different inference time ranges, with the corresponding color and marker schemes described in the legend boxes located at the left and right centers, respectively. Devices running Android version 14 (blue and red dashed curves) fall within the ultra-low-latency zone, while the device running Android 13 (yellow dashed curve) lies at the boundary between the low-latency and real-time response zones. Devices operating on Android versions below 10 (green dashed curve) are positioned within the real-time response zone.

**Figure 5:**
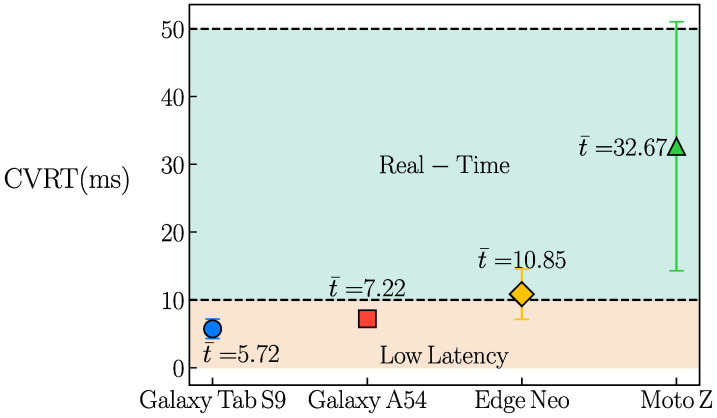
Computer vision response time (CVRT) for each tested mobile device.

The computer vision response time (CVRT), measured in milliseconds, is defined as the mean value μ obtained from the fitting process. Figure 5 presents the CVRT obtained for each mobile device. Vertical bars associated with each marker indicate the standard deviation $\sigma^{2}$ derived from the fit, representing the variability in inference time. For clarity, the CVRT values for each device are displayed in adjacent text boxes. All tested mobile devices exhibit an average computer vision response time below 50,ms, confirming real-time performance. Furthermore, as the Android version increases, the response shifts from the real-time zone to the ultra-low-latency zone, indicating that newer devices are less likely to experience any perceptible delay during computer vision-assisted interaction.

### 5.2. User Experience Evaluation

The user experience evaluation of the GECO platform aimed to assess the usability, accessibility and inclusivity of a computer vision-assisted gesture interface for smart home control. In the experiment, seventeen participants without any disabilities and an average age of 27 years (minimum of 22 and maximum of 40), were asked to interact with the GECO application, using gestures to navigate the interface and control IoT devices. None of the participants had any prior interaction with the platform, nor were they provided with any form of explanation or introductory guidance. During this group’s interaction with the GECO application, no procedural guidance or instructional steps were provided to the participants. Consequently, the second test was designed to evaluate the users’ capacity to operate and manipulate the platform autonomously, relying solely on its interface and interaction design. After completing the interaction, participants filled out a structured a custom questionnaire tailored to evaluate several aspects of the interface, including gesture understanding, visual feedback, recognition accuracy, intuitiveness, and perceived usefulness for specific user groups. The questionnaire employed a 10-point Likert scale, with higher scores indicating more favorable assessments.

**Figure 6:**
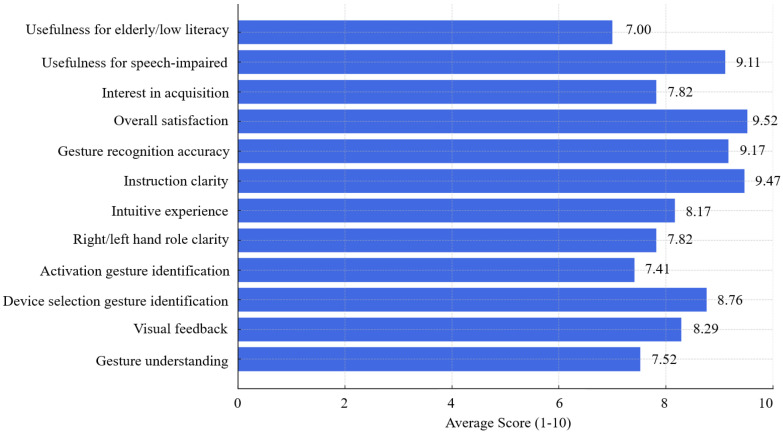
Average user scores from the GECO platform usability evaluation. Each blue bar represents the mean score (on a 1–10 scale) attributed by participants to a specific usability aspect of the gesture-based interface.

Figure 6 presents the results, indicating an overall positive user perception of the GECO interface. Most participants found the system intuitive, reporting high scores for gesture recognition accuracy (9.17) and overall satisfaction after learning the interface (9.52). The instructional content received a score of 9.47, highlighting the effectiveness of the integrated visual guidance system. The gesture-to-function mapping was perceived as effective, with selection gestures scoring 8.76 and activation gestures 7.41. Participants also recognized the system’s potential benefits for people with speech impairments (9.11) and considered it moderately suitable for elderly users or those with low technological literacy (7.00). Moreover, the perception of interaction indicated a medium cognitive load experienced by the participants during the experiment, as reflected by the gesture understanding and activation gesture identification scores of 7.52 and 7.41, respectively. Overall, the user experience survey demonstrates that the GECO platform achieves high usability and inclusivity. However, some scores suggest areas for improvement, particularly simplifying gesture roles for faster comprehension and enhancing accessibility for a broader range of users.

## 6. Conclusions

This paper presented GECO, a computer vision-assisted gesture control platform designed for smart home automation within IoT ecosystems. By combining markerless hand tracking with a structured interaction workflow, the system provides an intuitive and non-contact alternative to traditional control interfaces. GECO exemplifies the convergence of computer vision, embedded systems, and user-centered design principles, showing that lightweight AI models can operate efficiently on mobile devices while preserving real-time responsiveness. The user evaluation indicates high levels of perceived intuitiveness, precision, and relevance, confirming the potential of gesture interfaces to enhance accessibility and technological inclusion. Furthermore, performance assessments across multiple mobile platforms demonstrate GECO’s compatibility and scalability in real-world scenarios. Hence, GECO contributes to the growing body of research on context-aware, AI-driven human-computer interaction within smart environments. Its modular and standards-compliant design enables adaptation to diverse domains beyond home automation, including industrial IoT, assistive technologies, and smart public infrastructures. Future work includes integrating adaptive learning for personalized gesture modeling, exploring richer multimodal interfaces that jointly use gestures and speech, and conducting longitudinal studies to assess robustness across diverse user groups and environmental conditions.

## Figures and Tables

**Table 1:** Comparison of GECO with Related Works.

Aspect	Previous Works	GECO Contribution
Gesture Sensing	IMU-based wearable sensors [37,39] or specialized cameras/gloves [16,40].	Fully markerless vision-based sensing using standard mobile cameras and MediaPipe, no specialized hardware required.
Device Control Architecture	Device selection mainly, partial actuation [37]. External servers are often involved.	Full end-to-end IoT device control using private MQTT-based network, from gesture to actuation.
Gesture Recognition Technique	SVM [39], RVM [36], or CNNs with large datasets [18,41].	Lightweight MediaPipe hand landmark detection with real-time classification, no heavy retraining or large datasets required.
User Interaction Model	Single-hand gesture systems for both selection and command [15,18].	Two-hand, two-phase interaction (right hand for navigation, left hand for commands), improving usability and reducing errors.
Inclusivity Focus	Works focused on specific disabilities (e.g., deaf users [41], elderly users [20]).	Designed for a broad range of users, including the elderly, nonverbal, and non-technical users, promoting intuitive and accessible interaction.
Intensity/Analog Control	Most systems provide binary (on/off) commands [37,39,42].	Introduced continuous control (e.g., light dimming) using thumb-index angle computation for analog intensity settings.
Privacy and Local Processing	Often cloud-reliant solutions with external processing [21,42].	Fully private local processing: local Wi-Fi and MQTT network without external data transmission, enhancing security and privacy, and reducing latency.

**Table 2:** Commercial mobile devices used in the user experience experiment.

Device Model	Android Version
Galaxy Tab S9	Android 14.0
Galaxy A54	Android 14.0
Edge Neo	Android 13.0
Moto Z	Android 9.0

## Data Availability

There are no data related with this work.

## Abbreviations

The following abbreviations are used in this manuscript:

AI	Artificial Intelligence
AAL	Ambient Assisted Living
AIF	Average Inference Time
API	Application Programming Interface
ASL	American Sign Language
CNN	Convolutional Neural Network
CVRT	Computer Vision Response Time
DC	Direct Current
ECDF	Empirical Cumulative Distribution Function
FrFT	Fractional Fourier Transform
GECO	Gesture-Controlled Environment Controller
H-bridge	Half Bridge
IMU	Inertial Measurement Unit
IoT	Internet of Things
ITU-T	International Telecommunication Union—Telecommunication Standardization Sector
LPWAN	Low-Power Wide-Area Network
ML	Machine Learning
MQTT	Message Queuing Telemetry Transport
NLP	Natural Language Processing
NLU	Natural Language Understanding
PDF	Probability Density Function
RNN	Recurrent Neural Network
RVM	Relevance Vector Machine
SDK	Software Development Kit
SVM	Support Vector Machine
UWB	Ultra-Wideband
UI/UX	User Interface/User Experience
Wi-Fi	Wireless Fidelity
5G	Fifth Generation

## References

[1] Al-Fuqaha A., Guizani M., Mohammadi M., Aledhari M., Ayyash M. (2015). Internet of Things: A Survey on Enabling Technologies, Protocols, and Applications. IEEE Commun. Surv..

[2] de Carvalho J.G.A., da Conceição A.A., Ambrósio L.P., Fernandes F., Ramborger E.H.T., Aquino G.P., Boas E.C.V. (2024). Smart Lab: An IoT-centric Approach for Indoor Environment Automation. J. Commun. Inf. Syst..

[3] da Conceic’ão A.A., Ambrosio L.P., Leme T.R., Rosa A.C., Ramborger F.F., Aquino G.P., Boas E.C.V. (2022). Internet of things environment automation: A smart lab practical approach. Proceedings of the 2022 2nd International Conference on Information Technology and Education (ICIT&E).

[4] Chettri L., Bera R. (2019). A comprehensive survey on Internet of Things (IoT) toward 5G wireless systems. IEEE Internet Things J..

[5] Nikoukar A., Raza S., Poole A., Güneş M., Dezfouli B. (2018). Low-power wireless for the internet of things: Standards and applications. IEEE Access.

[6] Dhillon H.S., Huang H., Viswanathan H. (2017). Wide-area wireless communication challenges for the Internet of Things. IEEE Commun. Mag..

[7] Yang C., Liang P., Fu L., Cui G., Huang F., Teng F., Bangash Y.A. (2022). Using 5G in smart cities: A systematic mapping study. Intell. Syst. Appl..

[8] Bellini P., Nesi P., Pantaleo G. (2022). IoT-enabled smart cities: A review of concepts, frameworks and key technologies. Appl. Sci..

[9] Alavi A.H., Jiao P., Buttlar W.G., Lajnef N. (2018). Internet of Things-Enabled Smart Cities: State-of-the-Art and Future Trends. Measurement.

[10] Talari S., Shafie-Khah M., Siano P., Loia V., Tommasetti A., Catalão J.P. (2017). A review of smart cities based on the internet of things concept. Energies.

[11] Mehmood Y., Ahmad F., Yaqoob I., Adnane A., Imran M., Guizani S. (2017). Internet-of-things-based smart cities: Recent advances and challenges. IEEE Commun. Mag..

[12] Yin C., Xiong Z., Chen H., Wang J., Cooper D., David B. (2015). A literature survey on smart cities. Sci. China Inf. Sci..

[13] Gracias J.S., Parnell G.S., Specking E., Pohl E.A., Buchanan R. (2023). Smart cities—A structured literature review. Smart Cities.

[14] Allam Z., Dhunny Z.A. (2019). On big data, artificial intelligence and smart cities. Cities.

[15] Khan L.U., Yaqoob I., Tran N.H., Kazmi S.A., Dang T.N., Hong C.S. (2020). Edge-computing-enabled smart cities: A comprehensive survey. IEEE Internet Things J..

[16] Hajam S.S., Sofi S.A. (2021). IoT-Fog architectures in smart city applications: A survey. China Commun..

[17] Afonso M.H.F., Teixeira E.H., Cruz. M.R., Aquino G.P., Vilas Boas E.C. Vehicle and Plate Detection for Intelligent Transport Systems: Performance Evaluation of Models YOLOv5 and YOLOv8. Proceedings of the 2023 IEEE International Conference on Computing (ICOCO).

[18] Li J., Lin Y. IoT Home Automation—Smart homes and Internet of Things. Proceedings of the 2021 3rd International Academic Exchange Conference on Science and Technology Innovation (IAECST).

[19] Almusaed A., Yitmen I., Almssad A. (2023). Enhancing smart home design with AI models: A case study of living spaces implementation review. Energies.

[20] Guo X., Shen Z., Zhang Y., Wu T. (2019). Review on the application of artificial intelligence in smart homes. Smart Cities.

[21] Elkhalik W.A. (2023). AI-Driven Smart Homes: Challenges and Opportunities. J. Intell. Syst. Internet Things.

[22] Jiang L., Zhang L., Wang X. (2024). Integration of Cross-Computer Science and Architectural Design for the Elderly: AI for Smart Home. Proceedings of the International Conference on Inventive Communication and Computational Technologies.

[23] Azzedine D.E. (2024). AI-Powered Conversational Home Assistant for Elderly Care. Master’s Thesis.

[24] Periša M., Teskera P., Cvitić I., Grgurević I. (2025). Empowering People with Disabilities in Smart Homes Using Predictive Informing. Sensors.

[25] Mora-Sánchez O.B., López-Neri E., Cedillo-Elias E.J., Aceves-Martínez E., Larios V.M. (2021). Validation of IoT Infrastructure for the Construction of Smart Cities Solutions on Living Lab Platform. IEEE Trans. Eng. Manag..

[26] Negreiros I., Francisco A.C.C., Fengler F.H., Faria G., Pinto L.G.P., Tolotto M., Rogoschewski R.B., Romano R.R., Netto R.S. Smart Campus® as a Living Lab on Sustainability Indicators Monitoring. Proceedings of the 2020 IEEE International Smart Cities Conference (ISC2).

[27] Kadar M. Smart Learning Environment for the Development of Smart City Applications. Proceedings of the 2016 IEEE 8th International Conference on Intelligent Systems (IS).

[28] Knight N.J., Kanza S., Cruickshank D., Brocklesby W.S., Frey J.G. (2020). Talk2Lab: The Smart Lab of the Future. IEEE Internet Things J..

[29] Babu C.S., Purushothaman R., Anusha K., Sakthi S. (2023). Smart Gesture Controlled Systems Using IoT. AI-Based Digital Health Communication for Securing Assistive Systems.

[30] Harris M., Agoes A.S., Indriani (2021). Applying hand gesture recognition for user guide application using MediaPipe. Proceedings of the 2nd International Seminar of Science and Applied Technology (ISSAT 2021).

[31] Altayeb M. (2023). Hand gestures replicating robot arm based on mediapipe. Indones J. Electr. Eng. Inform..

[32] Torres W., Santos L., Melo G., Oliveira A., Nascimento P., Carvalho G., Neves T., Martins A., Araújo Í. (2024). A Framework for Real-Time Gestural Recognition and Augmented Reality for Industrial Applications. Sensors.

[33] Huda M.R., Ali M.L., Sadi M.S. (2025). Developing a real-time hand-gesture recognition technique for wheelchair control. PLoS ONE.

[34] Nguyen T.H., Ngo B.V., Nguyen T.N. (2025). Vision-Based Hand Gesture Recognition Using a YOLOv8n Model for the Navigation of a Smart Wheelchair. Electronics.

[35] Chandolikar N., Bondarde A., Bodade N., Bornar V., Borkar D., Borbande S., Borkar P. (2024). Hand Gesture Controlled Wheelchair. Proceedings of the 2024 International Conference on Artificial Intelligence and Quantum Computation-Based Sensor Application (ICAIQSA).

[36] Xie H., Zhao Y. (2025). Advanced Gesture Recognition Method Based on Fractional Fourier Transform and Relevance Vector Machine for Smart Home Appliances. Comput. Animat. Virtual Worlds.

[37] Alanwar A., Alzantot M., Ho B.J., Martin P., Srivastava M. Selecon: Scalable iot device selection and control using hand gestures. Proceedings of the Second International Conference on Internet-of-Things Design and Implementation.

[38] Panagiotou C., Faliagka E., Antonopoulos C.P., Voros N. (2025). Multidisciplinary ML Techniques on Gesture Recognition for People with Disabilities in a Smart Home Environment. AI.

[39] Ameliasari M., Putrada A.G., Pahlevi R.R. (2021). An evaluation of svm in hand gesture detection using imu-based smartwatches for smart lighting control. J. Infotel.

[40] Alabdullah B.I., Ansar H., Mudawi N.A., Alazeb A., Alshahrani A., Alotaibi S.S., Jalal A. (2023). Smart home automation-based hand gesture recognition using feature fusion and recurrent neural network. Sensors.

[41] Fatima B., Mushtaq B., Iqbal M.A., Ahmed A. (2024). IoT-based Smart Home Automation Using Gesture Control and Machine Learning for Individuals with Auditory Challenges. ICCK Trans. Internet Things.

[42] Yang C.Y., Lin Y.N., Wang S.K., Shen V.R., Tung Y.C., Shen F.H., Huang C.H. (2023). Smart control of home appliances using hand gesture recognition in an IoT-enabled system. Appl. Artif. Intell..

[43] Dinh D.L., Kim J.T., Kim T.S. (2014). Hand gesture recognition and interface via a depth imaging sensor for smart home appliances. Energy Procedia.

[44] Alemuda F., Lin F.J. (2017). Gesture-based control in a smart home environment. Proceedings of the 2017 IEEE International Conference on Internet of Things (iThings) and IEEE Green Computing and Communications (GreenCom) and IEEE Cyber, Physical and Social Computing (CPSCom) and IEEE Smart Data (SmartData).

[45] Zhu H., Dong E., Xu M., Lv H., Wu F. (2024). Commodity Wi-Fi-Based Wireless Sensing Advancements over the Past Five Years. Sensors.

[46] Wang M., Huang J., Zhang X., Liu Z., Li M., Zhao P., Yan H., Sun X., Dong M. (2025). Target-Oriented WiFi Sensing for Respiratory Healthcare: From Indiscriminate Perception to In-Area Sensing. IEEE Netw..

[47] Kirimtat A., Krejcar O., Kertesz A., Tasgetiren M.F. (2020). Future Trends and Current State of Smart City Concepts: A Survey. IEEE Access.

[48] Farooq M.S., Riaz S., Abid A., Abid K., Naeem M.A. (2019). A Survey on the Role of IoT in Agriculture for the Implementation of Smart Farming. IEEE Access.

[49] Wollschlaeger M., Sauter T., Jasperneite J. (2017). The Future of Industrial Communication: Automation Networks in the Era of the Internet of Things and Industry 4.0. IEEE Ind. Electron. Mag..

[50] Zhang F., Bazarevsky V., Vakunov A., Tkachenka A., Sung G., Chang C.L., Grundmann M. (2020). Mediapipe hands: On-device real-time hand tracking. arXiv.

[51] Simon T., Joo H., Matthews I., Sheikh Y. (2017). Hand Keypoint Detection in Single Images using Multiview Bootstrapping. arXiv.

[52] Jiang T., Lu P., Zhang L., Ma N., Han R., Lyu C., Li Y., Chen K. (2023). RTMPose: Real-Time Multi-Person Pose Estimation based on MMPose. arXiv.

[53] Cai S., Xu H., Cai W., Mo Y., Wei L. (2025). A human pose estimation network based on YOLOv8 framework with efficient multi-scale receptive field and expanded feature pyramid network. Sci. Rep..

[54] Bazarevsky V., Kartynnik Y., Vakunov A., Raveendran K., Grundmann M. (2019). Blazeface: Sub-millisecond neural face detection on mobile gpus. arXiv.

[55] Google Model Card Hand Tracking (Lite/Full) with Fairness Oct 2021. https://storage.googleapis.com/mediapipe-assets/Model%20Card%20Hand%20Tracking%20(Lite_Full)%20with%20Fairness%20Oct%202021.pdf.

[56] Google (2022). Model Card Gesture Classification with Fairness 2022. https://storage.googleapis.com/mediapipe-assets/gesture_recognizer/model_card_hand_gesture_classification_with_faireness_2022.pdf.

[57] Google (2022). Hand Land Mark Medipipe. https://ai.google.dev/edge/mediapipe/solutions/vision/hand_landmarker.

[58] Virtanen P., Gommers R., Oliphant T.E., Haberland M., Reddy T., Cournapeau D., Burovski E., Peterson P., Weckesser W., Bright J. (2020). SciPy 1.0: Fundamental algorithms for scientific computing in Python. Nat. Methods.

